# Comparison between [^68^Ga]Ga-FAPI-46 and [^18^F]FDG PET/CT uptake in luminal-like vs. HER2-positive breast cancer

**DOI:** 10.1186/s13550-026-01390-3

**Published:** 2026-03-13

**Authors:** Alina Toni Küper, Sofia Carrilho Vaz, Kim Magaly Pabst, Ieva Ciuciulkaite, Ilektra Antonia Mavroeidi, Rainer Hamacher, Anja Welt, Noah Hammersen, Pedro Fragoso Costa, Loic Djaileb, Robert Seifert, Lale Umutlu, Martin Schuler, Ken Herrmann, Wolfgang Peter Fendler, Sherko Kümmel, David Kersting

**Affiliations:** 1 Department of Nuclear Medicine and German Cancer Consortium (DKTK), University Hospital Essen, University of Duisburg-Essen, Essen, Germany; 2 Department of Nuclear Medicine and Radiopharmacology, Champalimaud Clinical Center, Champalimaud Foundation, Lisbon, Portugal; 3 Department of Radiology, Leiden University Medical Center, Leiden, The Netherlands; 4 Department of Medical Oncology, NCT site, University Hospital Essen, West German Cancer Center, University of Duisburg-Essen, Essen, Germany; 5 German Cancer Consortium (DKTK), Partner Site University Hospital Essen, Essen, Germany; 6 Nuclear Medicine Department, LRB, CHU Grenoble Alpes, INSERM, Université Grenoble Alpes, Grenoble, France; 7 Department of Nuclear Medicine, University Hospital Bern, University of Bern, Bern, Switzerland; 8 Institute of Interventional and Diagnostic Radiology and Neuroradiology, University Hospital Essen, University of Duisburg-Essen, Essen, Germany; 9 Breast Unit Essen, Kliniken Essen-Mitte, Essen, Germany

**Keywords:** Breast cancer, PET, FDG, FAPI, Pan-tumour imaging, Hormone receptors, Luminal-like, HER2, Theranostics

## Abstract

**Background:**

Fibroblast activation protein (FAP)–targeted tracers have emerged as promising agents for breast cancer imaging, with recent studies demonstrating PET performance comparable to or surpassing, that of [^18^F]FDG. Nevertheless, data comparing [^68^Ga]Ga-FAPI-46 and [^18^F]FDG uptake in hormone receptor and/or HER2–positive breast cancer (luminal-like vs HER2-positive) remain scarce. Aim of this study was to investigate the diagnostic performance of [^68^Ga]Ga-FAPI-46 versus [^18^F]FDG PET/CT in patients with hormone-receptor and/or HER2-positive breast cancer, and to evaluate the uptake of both tracers stratified by molecular subtypes (luminal-like vs HER2-positive). A sub-analysis of a prospective observational trial (NCT04571086) was conducted. Patients with histologically confirmed, hormone receptor- and/or HER2-positive breast cancer who underwent whole-body [^68^Ga]Ga-FAPI-46 and [^18^F]FDG PET/CT in the same week for initial staging or follow-up were included. [^68^Ga]Ga-FAPI-46 or [^18^F]FDG PET-positive lesions were defined as visually increased lesion uptake compared to adjacent organ background. Semi-automatic segmentation was performed to determine SUVmax, SUVpeak, TLRpeak, total number of lesions, total tumour volume, and total tumour SUV mean. Data were compared between molecular subtypes (luminal-like vs HER2-positive).

**Results:**

Thirteen patients were included. Overall, the detection performance was comparable between [^68^Ga]Ga-FAPI-46 and [^18^F]FDG PET. The semi-quantitative analysis showed comparable mean uptake values in breast cancer lesions on [^68^Ga]Ga-FAPI-46 and [^18^F]FDG PET/CT (SUV_max_: 13.4 vs. 12.9; TLR_peak_: 5.6 vs. 4.5) and revealed no significant differences in the median lesion count (4.5 vs. 5), mean total tumour volume (71.5 vs. 73.2 mL), or mean total tumour SUV_mean_ (5.4 vs. 5.4). No substantial differences between molecular subtypes (luminal-like vs. HER2-positive) were observed.

**Conclusion:**

In this small exploratory cohort, comparable uptake patterns in [^68^Ga]Ga-FAPI-46- and [^18^F]FDG-positive breast cancer lesions were observed across subtypes, underscoring the potential of [^68^Ga]Ga-FAPI-46 as a versatile imaging tool. Future studies in larger cohorts are warranted to explore the potential of FAP-targeted theranostics in different breast cancer subtypes.

**Trial registration:**

68-Ga-FAPI-PET for Tumor Detection: A Prospective Observational Trial, NCT04571086, 09-15-2020, https://clinicaltrials.gov/study/NCT04571086.

**Supplementary Information:**

The online version contains supplementary material available at 10.1186/s13550-026-01390-3.

## Introduction

Breast cancer is the most common cancer in women worldwide. In 2022 it was the second most frequently diagnosed cancer and the leading cause of cancer-related death in women [1]. By 2040, breast cancer is predicted to increase to over 3 million new cases and 1 million deaths per year, being a major health concern [2].

Breast cancer is a heterogenous disease, characterized by diverse biological features and clinical presentations. The main molecular subtypes are Luminal A-like in 50/60% (hormone receptor (HR)-positive, HER2-negative and low-grade/low proliferation), Luminal B-like in 15/20% (HR-positive, HER2-negative and high-grade/high proliferation) and HER2-positive in 10/15% of cases (HR-positive or negative and HER2-positive) [3, 4]. Breast cancer is usually staged according to the Union for International Cancer Control (UICC) in primary tumour, nodal status and distant metastases (TNM) classification [5].

Imaging is essential for staging. Mammography, sonography, and MRI are often used for locoregional assessment. Despite significant developments and promising results from molecular imaging modalities, they are mainly used in ambiguous cases or systemic staging in advanced disease [6]. Although [^18^F]fluorodeoxyglucose (FDG) remains the most widely used positron emission tomography (PET) tracer for systemic staging in breast cancer, its performance is constrained by several well-recognised limitations. These include reduced sensitivity for small lesions, micrometastatic disease, hormone receptor-positive tumours, and lobular subtype. This limitation is well documented for luminal A and, to a lesser extent, luminal B tumours, which show lower [^18^F]FDG-avidity than HER2-positive or triple-negative cancers. FDG uptake is also prone to false positive results, particularly after biopsy or in benign breast conditions such as infection, fibroadenoma, or ductal adenoma [7, 8].

Recent literature has demonstrated promising results with more specific tracers such as [^18^F]fluoroestradiol (FES) for estrogen receptor-positive tumours and [^89^Zr]trastuzumab for HER2-positive disease [9, 10]. Nevertheless, these tracers are not widely available and seem particularly useful in selecting patients for targeted therapy [11, 12].

In contrast, fibroblast activation protein inhibitor (FAPI) based tracers (e.g., [^68^Ga]Ga-FAPI-46) are emerging compounds that may provide advantageous imaging properties across all breast cancer subtypes, as they visualise cancer-associated fibroblasts (CAFs) and thereby reflect stromal remodelling and desmoplasia [13, 14]. Fibroblast activation protein (FAP) is highly expressed on the membrane of CAFs, which is the most abundant component of the tumour microenvironment, and can contribute to tumour angiogenesis, migration, and invasion of tumour cells [15, 16]. Several studies have demonstrated the benefit of using FAP tracers for the detection of breast cancer lesions, reporting potentially higher sensitivity, maximum standardized uptake values (SUV_max_), and tumour-to-background ratios compared to [^18^F]FDG [14, 17–20]. Moreover, FAP tracers were superior to [^18^F]FDG in in identifying lymph node, hepatic, bone, and brain metastases [14, 21]. There are increasing expectations about the use of FAP tracers as a pan-tumour marker with theranostic potential, including in breast cancer [13, 22–25].

Despite a growing number of studies comparing the diagnostic performance of [^18^F]FDG and [^68^Ga]Ga-FAP tracers in breast cancer, most investigations assess breast cancer as a single entity-without stratifying by hormone receptors or HER2 expression. Exceptions are limited to a few studies specifically addressing the lobular subtype [26, 27] and a small number focusing on triple-negative breast cancer [28, 29]. Consequently, data comparing [^68^Ga]Ga-FAPI-46 and [^18^F]FDG uptake in luminal-like and HER2-positive breast cancer remain scarce.

The aim of this study was to investigate the diagnostic performance of [^68^Ga]Ga-FAPI-46 versus [^18^F]FDG PET/CT in patients with hormone receptor- and/or HER2-positive positive breast cancer. As a secondary objective, we evaluated [^68^Ga]Ga-FAPI-46 and [^18^F]FDG PET/CT uptake stratified by molecular subtypes (luminal-like vs. HER2-positive).

## Methods

### Patients and ethics

A sub-analysis of the prospective University Hospital Essen [^68^Ga]Ga-FAPI-46 PET observational trial (NCT04571086) was conducted. Patients with histologically confirmed, hormone receptor- and/or HER2-positive breast cancer who underwent clinically indicated whole-body [^18^F]FDG and [^68^Ga]Ga-FAPI-46 PET/CT in the same week for initial diagnostic workup or follow-up staging between October 2021 and April 2025 were consecutively included. The clinically requested FAPI examination routinely entailed complementary [^18^F]FDG PET/CT imaging according to institutional standards. Patient referrals were made by the treating gynaecologic oncologists. If patients were scanned with [^68^Ga]Ga-FAPI-46 PET/CT more than once, only the first paired examination was included. Patients with triple negative breast cancer and those without any [^68^Ga]Ga-FAPI-46 or [^18^F]FDG positive lesions were excluded from further analysis. Patients were categorized according to hormone receptor and HER2 expression into luminal A-like, luminal-B like and HER2-positive (including the HER2-enriched subtype). For subsequent analyses, luminal A-like and luminal B-like tumours were combined into a single hormone receptor-positive group due to the limited number of patients in each individual subtype. Written informed consent for study participation and to undergo clinical PET examinations were obtained from all patients. All investigations were conducted in accordance with the Declaration of Helsinki and national regulations. The local institutional ethics committee (University of Duisburg–Essen, medical faculty) approved the study (ethics protocol permits 19–8991-BO and 20–9485-BO).

### [^68^Ga]Ga-FAPI-46 and [^18^F]FDG PET/CT imaging

PET/CT images were acquired on a Biograph Vision 600 or a Biograph mCT PET/CT system (both Siemens Healthineers, Erlangen, Germany). The mean [^68^Ga]Ga-FAPI-46 administered activity was 104 MBq (62–144) and the mean uptake time was 20 min (10–99). The mean [^18^F]FDG administered activity was 231 MBq (82–377) and the mean uptake time was 101 min (53–305). According to institutional protocols, patients underwent a contrast-enhanced whole-body CT scan prior to PET, provided such an exam had not already been performed within the previous four weeks. If a recent CT was available, a low-dose, non-contrast CT was instead acquired and used for attenuation correction and anatomical localization of PET uptake. PET data were acquired and PET images were reconstructed according to our standard institutional protocols [30].

### PET image interpretation

All PET images were analyzed separately by three nuclear medicine physicians using Syngo.via software (version VB80D; Siemens Healthineers). In cases of divergent findings, the images were reexamined to establish a consensus. Nuclear medicine physicians confirmed imaging quality. Patient-based and region-based analysis for [^68^Ga]Ga-FAPI-46 or [^18^F]FDG PET-positive lesions were reported for each patient separately. Six different anatomic categories derived from the UICC TNM classification for breast cancer were defined [5]: primary tumour, regional lymph node metastases, distant lymph node metastases, liver metastases, bone metastases and other distant metastases.

Lesions were classified as metastatic if they showed abnormal radiotracer uptake in a typical metastatic location or when uptake was low but an anatomically matching abnormality was detectable on CT. In case of unclear interpretation of lesion uptake, clinical information, blood test and other imaging modalities were considered, as biopsy was not performed. The final interpretation of the PET/CT examination was subsequently reviewed in an interdisciplinary tumour board to ensure consensus assessment. [^68^Ga]Ga-FAPI-46-/[^18^F]FDG-positivity was defined as visually markedly increased lesion uptake compared to adjacent organ background. Brain metastases were described but not included into quantitative analysis due to the high physiologic cerebral [^18^F]FDG uptake, which limits lesion identification. Additionally, the skull is not included in the standard scan field of view of contrast-enhanced PET protocols for oncologic PET/CT imaging at our institution.

### Lesion analysis and quantification

Semi-automatic segmentation of all [^68^Ga]Ga-FAPI-46- and/or [^18^F]FDG PET-positive lesions per patient was performed, using a PERCIST-like approach considering a liver-specific threshold defined as lesion peak standard uptake value (SUV_peak_) that was required to be at least (1.5 x SUV_mean_ liver + 2 x standard deviation (SD) of SUV liver) [31]. Liver SUV_mean_ values were determined in a spherical volume-of-interest of 14 mL volume (3-cm diameter) in the right liver lobe as suggested in PERCIST 1.0. Lesion boundaries were determined using a 41% isocontour approach [32]. Small lesions with a volume below 0.5 ml were excluded. Additional foci with lower SUV values were manually included when required, while physiologic uptake was manually excluded. Lesion SUV_max_, SUV_peak_, tumour-to-liver ratio _peak_ (TLR_peak_), total number of lesions, total tumour volume (sum of the volumes of all [^68^Ga]Ga-FAPI-46 or [^18^F]FDG PET-positive lesions), and total tumour SUV_mean_ (SUV_mean_ in the entire total tumour volume) were determined.

### Statistics

All statistical analyses were conducted using IBM Statistical Package for the Social Sciences (SPSS) Statistics software (version 29.0.0, IBM Corp., Armonk, N.Y., USA). Mean values were presented as mean ± standard deviation (SD). The Shapiro-Wilk test was applied to verify normal distribution. To compare [^68^Ga]Ga-FAPI-46 and [^18^F]FDG uptake (analyzing the total number of detected lesions, total tumour volume, and mean global SUV_mean_), Kruskal-Wallis Test was employed. To compare [^68^Ga]Ga-FAPI-46 and [^18^F]FDG uptake in each location of the luminal-like and HER2-positive groups (analyzing the SUV_max_ and TLR_peak_), the independent samples Mann-Whitney U Test was used. P-values (P) < 0.05 were considered statistically significant. The SUV_max_ and TLR_peak_ percentage differences between [^68^Ga]Ga-FAPI-46 and [^18^F]FDG uptake were calculated for each involved region and sub-category (luminal-like or HER2-positive).

## Results

### Patient and tumour characteristics

Thirteen women were included, the mean age was 57 years (ranging from 34 to 74 years). All patient except one underwent both [^68^Ga]Ga-FAPI-46 and [^18^F]FDG PET scans on the same day. In one case, [^68^Ga]Ga-FAPI-46 PET was conducted seven days before [^18^F]FDG PET for logistical reasons, with no invasive procedures or treatments undertaken between the examinations. In two patients, both PET/CT examinations were performed at baseline staging; in all other patients, scans were acquired during follow-up due to rising tumour markers or clinical-/imaging based suspicion of disease progression or resistance to therapy. The majority of patients had no special type breast cancer (8/13) and moderately (7/13) or poorly differentiated (6/13) cancer. One half had luminal-like breast cancer and the other half had HER2-positive breast cancer (detailed information about receptor status, HER2 and Ki-67 per patient is provided in Table 1 from the supplementary material). At baseline staging, four patients were classified UICC 4 and three patients UICC 1. In the majority of patients (10/13), tumour metastases were already known before the PET scan. Most patients were submitted to surgery and received systemic therapy before performing PET/CT (Table 1).

**Table 1:** Patient and breast cancer characteristics

Mean age (min-max)	57 (34–74)
**Subtype**	
No special type	8
Lobular	4
NA	1
**Hormone Receptors & HER2 expression**	
Luminal A-like	2
Luminal B-like	4
HER2-positive	6
Luminal A/HER2	1 (*)
**Differentiation grade**	
G1	0
G2	7
G3	6
**UICC staging at baseline**	
1	3
2a	2
2b	2
3c	2
4	4
**Known metastasis when performing PET**	
No metastatic disease	3
Metastatic disease	10
**Treatment**	
Naive	3
Breast surgery	8
Targeted therapy	9
Chemotherapy	6
Adjuvant radiation therapy	5
Additional radiation therapy (**)	4

(*) bilateral breast cancer (luminal A-like on the left side and HER2-positive on the right side).

(**) due to relapse in the axilla and chest wall

### [^68^Ga]Ga-FAPI-46 and [^18^F]FDG PET detection performance

**Fig. 1:**
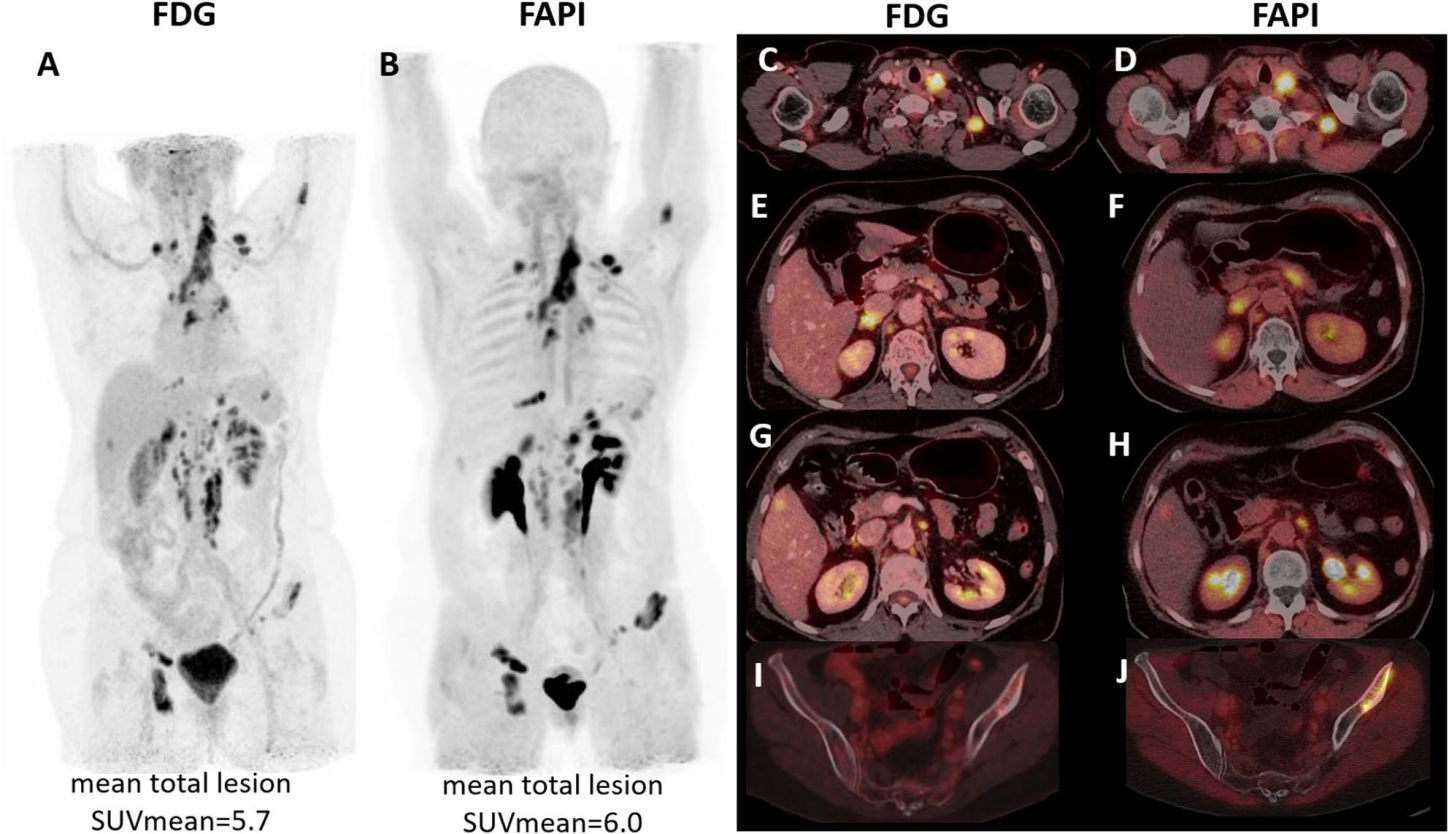
Comparable detection performance of [^18^F]FDG and [^68^Ga]Ga-FAPI-46 PET/CT. 74 years old woman diagnosed with left-sided ductal luminal B-like breast cancer (UICC stage 2a). PET/CT showed pathologic uptake in local lymph nodes (SUV_max_ [^18^F]FDG 11.0 vs. [^68^Ga]Ga-FAPI-46 10.5 - **C**&**D**), liver (SUV_max_ [^18^F]FDG 5.3 vs. [^68^Ga]Ga-FAPI-46 3.1), adrenal gland (SUV_max_ [^18^F]FDG 11.4 vs. [^68^Ga]Ga-FAPI-46 8.6- **E**&**F**), distant lymph nodes (SUV_max_ [^18^F]FDG 12.7 vs. [^68^Ga]Ga-FAPI-46 14.2 - **G**&**H**), bone (SUV_max_ [^18^F]FDG 13.9 vs. [^68^Ga]Ga-FAPI-46 12.2 - **I**&**J**), peritoneal (SUV_max_ [^18^F]FDG 8.9 vs. [^68^Ga]Ga-FAPI-46 10.5), and leptomeningeal metastasis (SUV_max_ [^68^Ga]Ga-FAPI-46 4.3, head not included in the [^18^F]FDG PET/CT FOV). Overall lesions presented similar [^18^F]FDG and [^68^Ga]Ga-FAPI-46 uptake (mean total lesion SUV_mean_ on [^18^F]FDG was 5.7 and [^68^Ga]Ga-FAPI-46 was 6.0). **A**&**B** - PET maximum intensity projection; **C**-**J** - PET/CT fused transaxial plans

Overall, the detection performance was comparable between [^68^Ga]Ga-FAPI-46 and [^18^F]FDG PET with [^68^Ga]Ga-FAPI-46 PET showing slight advantages for detection of distant metastases. In detail, uptake in the breast region was observed on both [^68^Ga]Ga-FAPI-46 and [^18^F]FDG PET/CT in five patients. Both modalities detected local lymph node involvement in three cases. In addition, [^68^Ga]Ga-FAPI-46 PET/CT revealed two further lesions, while [^18^F]FDG PET/CT identified three additional lesions. Distant lymph node metastases were visualized in three patients on both scans, and in two additional patients with [^68^Ga]Ga-FAPI-46 PET/CT only. Bone metastases showed concordant uptake in five patients. Additional metastatic sites with uptake on both PET/CT scans included the lung, liver, adrenal glands, and soft tissue in six patients. Among four patients with detected peritoneal metastases, one showed uptake exclusively on [^68^Ga]Ga-FAPI-46 PET/CT despite the presence of a morphological correlate on CT. In two patients, leptomeningeal and brain metastases were visible on [^68^Ga]Ga-FAPI-46 PET/CT. These findings could not be assessed on [^18^F]FDG PET/CT, as the head was not included in the scan range. Figure 1 illustrates a representative case demonstrating comparable detection performance between [^68^Ga]Ga-FAPI-46 and [^18^F]FDG PET/CT.

In the semi-quantitative analysis, breast cancer lesions showed comparable mean SUV_max_ and TLR_peak_ values on [^68^Ga]Ga-FAPI-46 and [^18^F]FDG PET/CT (SUV_max_: 13.4 ± 4.8 vs. 12.9 ± 14.2; TLR_peak_: 5.6 ± 3.2 vs. 4.5 ± 5.4, respectively) (Table 2). Across the entire cohort, no significant differences were observed between the tracers regarding median lesion count (n: 4.5 vs. 5), mean total tumour volume (71.5 ± 87.2 mL vs. 73.2 ± 124.4 mL), or mean total tumour SUV_mean_ (5.4 ± 2.7 vs. 5.4 ± 6.3) (Table 2; Fig. 1).

**Fig. 2:**
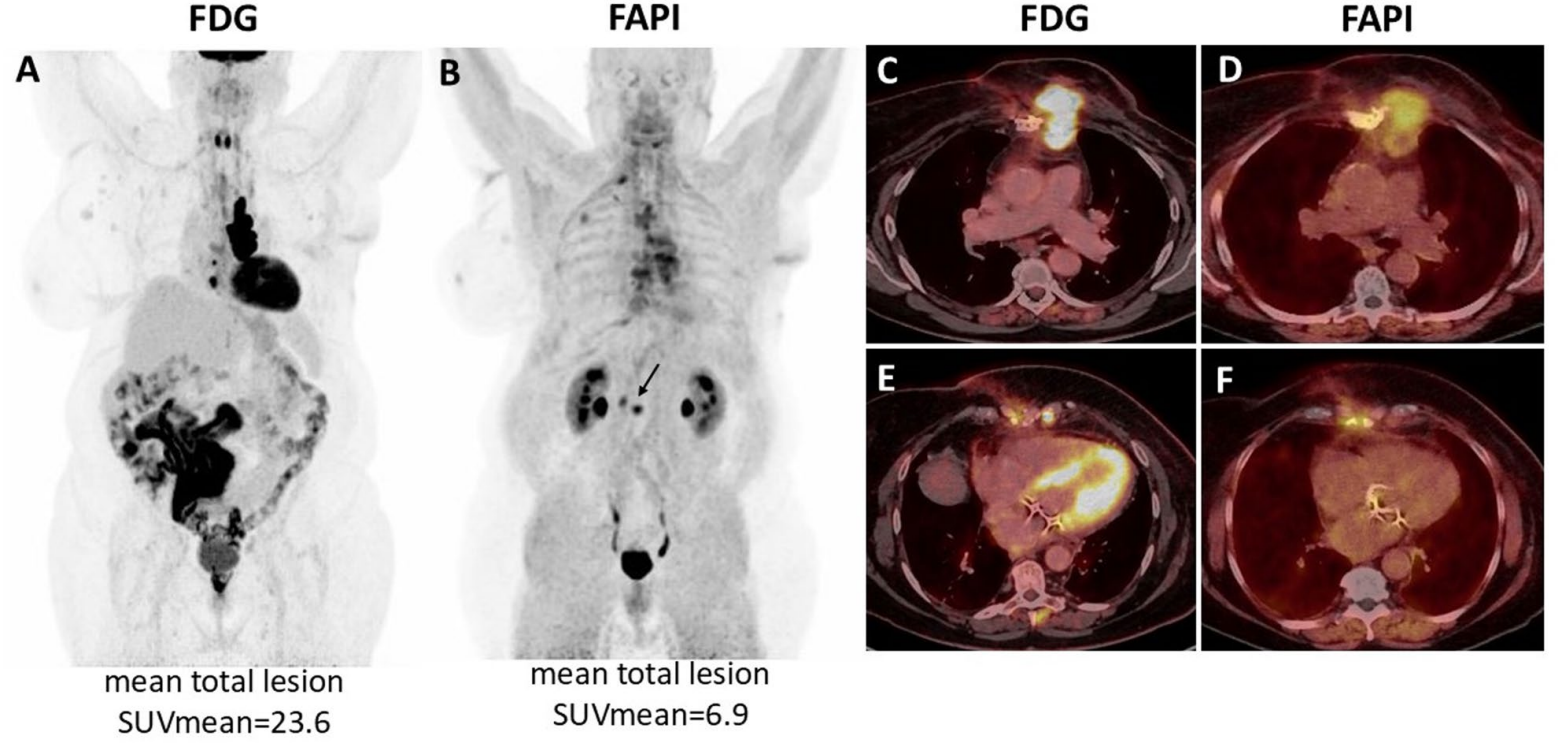
Superior detection performance of [^18^F]FDG PET/CT, compared to [^68^Ga]Ga-FAPI-46 PET/CT. 65 years old woman diagnosed with left-sided ductal luminal B-like breast cancer (UICC stage 3c). PET/CT showed abnormal uptake in a soft tissue mass involving the left para-sternal region of the thoracic wall (SUV_max_ [^18^F]FDG 42.3 vs. [^68^Ga]Ga-FAPI-46 7.6 - **C**&**D**), internal mammary lymph nodes (SUV_max_ [^18^F]FDG 18.9 vs. no [^68^Ga]Ga-FAPI-46 uptake - **E**&**F**), and bone (SUV_max_ [^18^F]FDG 4.1 vs. [^68^Ga]Ga-FAPI-46 9.8 (arrow in **B**)). Overall lesions presented higher [^18^F]FDG uptake than [^68^Ga]Ga-FAPI-46 uptake (mean total lesion SUV_mean_ on [^18^F]FDG 23.6 vs. [^68^Ga]Ga-FAPI-46 6.9). **A**&**B** - PET maximum intensity projection; **C**-**F** - PET/CT fused transaxial plans

Higher [^68^Ga]Ga-FAPI-46 than [^18^F]FDG uptake was observed in bone metastases (SUV_max_ 13.1 ± 6.4 vs. 8.5 ± 5.5 and TLR_peak_ 6.3 ± 5.8 vs. 2.0 ± 1.6). In a single patient with soft tissue metastasis, [^18^F]FDG uptake was higher than [^68^Ga]Ga-FAPI-46 (SUV_max_ 42.3 vs. 7.6 and TLR_peak_ 14.0 vs. 3.2) (Fig. 2).

**Fig. 3:**
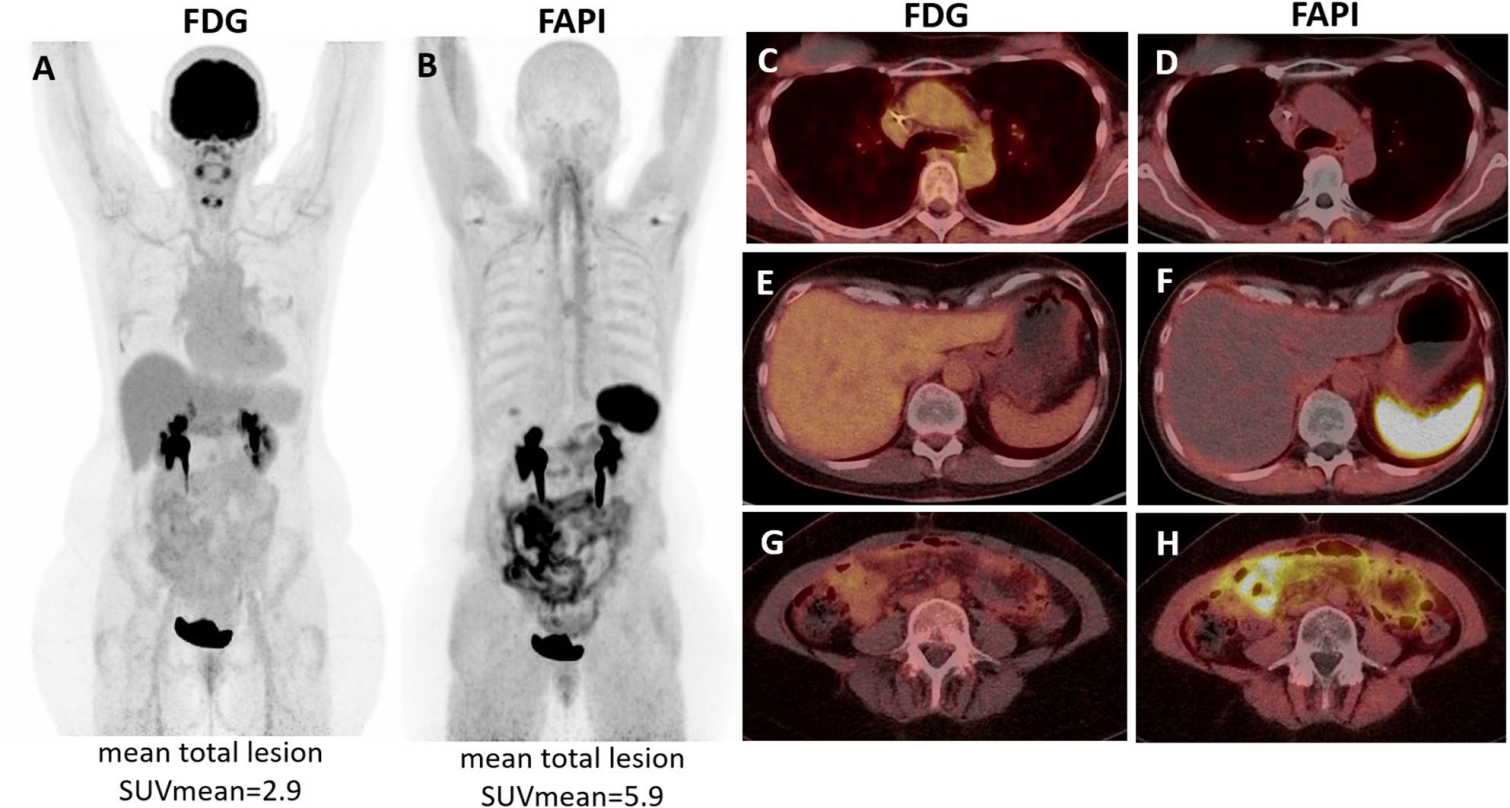
Superior detection performance of [^68^Ga]Ga-FAPI-46 PET/CT, compared to [^18^F]FDG PET/CT. 54 years old woman diagnosed with right-sited lobular luminal A-like breast cancer (UICC stage 2a). PET/CT showed pathologic uptake in distant lymph nodes (at the mediastinum SUV_max_ [^68^Ga]Ga-FAPI-46 2.4, no [^18^F]FDG uptake - **C**&**D**) and peritoneal metastases (SUV_max_ [^68^Ga]Ga-FAPI-46 14 vs. [^18^F]FDG 4 - **G**&**H**). Overall, lesions presented higher [^68^Ga]Ga-FAPI-46 uptake than [^18^F]FDG (mean total lesion SUV_mean_ on [^68^Ga]Ga-FAPI-46 5.9 vs. [^18^F]FDG 2.9). Intense diffuse [^68^Ga]Ga-FAPI-46 spleen uptake, probably therapy-related is observed (**F**). Seven months before PET, the patient had been submitted to abdominal surgery due to ileus and ascites, in which peritoneal carcinomatosis was diagnosed. **A**&**B** - PET maximum intensity projection; **C**-**H** - PET/CT fused transaxial plans

Generally, TLR was higher in [^68^Ga]Ga-FAPI-46 than [^18^F]FDG, particularly in local lymph nodes (6.1 ± 5.2 vs. 1.2 ± 1.3) and peritoneal metastases (7.7 vs. 1.7) (Fig. 3), as [^68^Ga]Ga-FAPI-46 showed lower liver uptake than [^18^F]FDG.

**Table 2:** [^68^Ga]Ga-FAPI-46 and [^18^F]FDG uptake per involved region

Involved regions		[^68^Ga]Ga-FAPI-46			[^18^F]FDG			Exclusive FAPI/FDG uptake
		Nº pts	SUV_max_	TLR_peak_	Nº pts	SUV_max_	TLR_peak_	
Breast		5	13.4 ± 4.8	5.6 ± 3.2	5	12.9 ± 14.2	4.5 ± 5.4	
Local lymph nodes		5	9.1 ± 6.9	**2.9 ± 1.8**	6	7.5 ± 6.5	**1.2 ± 1.3**	2 FAPI only 3 FDG only
M1	Distant lymph nodes	5	7.4 ± 4.6	3.3 ± 2.9	3	9.9 ± 5.1	2.6 ± 1.8	2 FAPI only
	Bone	5	**13.1 ± 6.4**	**6.3 ± 5.8**	5	**8.5 ± 5.5**	**2.0 ± 1.6**	
	Peritoneum	4	8.2 ± 5.3	**7.7 ± 5.4**	3	6.5 ± 2.5	**1.7 ± 0.7**	1 FAPI only
	Lung	1	5.5	2.2	1	6.9	1.2	
	Liver	1	3.1	1.6	1	5.3	3.8	
	Soft tissue	1	**7.6**	**3.2**	1	**42.3**	**14.0**	
	Adrenal gland	1	8.6	4.9	1	11.4	3.5	
								p-value
Nº of lesions		14.7 ± 20.0			18.9 ± 45.6			0.462
Total tumour volume		71.5 ± 87.2 mL			73.2 ± 124.4 mL			0.440
Total tumour SUV_mean_		5.4 ± 2.7			5.4 ± 6.3			0.355

Data are presented as mean ± SD (median and min-max are provided in supplementary material: supplementary Table 2). Bold highlight percentage difference between [^68^Ga]Ga-FAPI-46 and [^18^F]FDG uptake ≥ 50% for each involved according to the expression of hormone receptors (supplementary material: supplementary Table 4).

### [^68^Ga]Ga-FAPI-46 and [^18^F]FDG PET detection performance across molecular subtypes

One patient presenting bilateral breast cancer (luminal-like on the left, HER2-positive on the right side; Table 1) was excluded from this sub-analysis, resulting in 12 evaluable patients. In general, no substantial differences between detection performances and uptake values of [^68^Ga]Ga-FAPI-46 and [^18^F]FDG across luminal-like and HER2-positive breast cancer was observed (Table 3).

Most metastatic lesions were observed in patients with luminal-like breast cancer. In the luminal-like sub-group, [^18^F]FDG showed higher uptake than [^68^Ga]Ga-FAPI-46 in breast lesions (SUV_max_: 20.8 ± 23.3 vs. 9.6 ± 0.8; TLR_peak_: 7.5 ± 9.2 vs. 4.1 ± 0.1) and in a single soft tissue metastasis (SUV_max_: 42.3 vs. 7.6; TLR_peak_: 14.0 vs. 3.2). Uptake in other distant metastases was comparable between [^68^Ga]Ga-FAPI-46 and [^18^F]FDG PET (Table 3).

**Table 3:** Comparison of [^68^Ga]Ga-FAPI-46 and [^18^F]FDG uptake per involved region according to the expression of hormone receptors (luminal-like vs. HER2-positive)

Involved regions		[^68^Ga]Ga-FAPI-46 uptake						[^18^F]FDG uptake						
		Luminal SUV_max_	HER2 SUV_max_	*p*-value	Luminal TLR_peak_	HER2 TLR_peak_	*p*-value	Luminal SUV_max_		HER2 SUV_max_	*p*-value	Luminal TLR_peak_	HER2 TLR_peak_	*p*-value
Breast		**9.6 ± 0.8**	15.8 ± 4.8	0.2	**4.1 ± 0.1**	6.6 ± 4.1	0.8	**20.8 ± 23.3**		7.7 ± 5.5	1.0	**7.5 ± 9.2**	2.5 ± 0.7	1.0
Local lymph nodes		10.5	10.1 ± 9.1	1.0	**5.1**	2.8 ± 1.8	1.0	8.9 ± 0.8		4.8 ± 1.9	1.0	**1.1 ± 1.5**	1.5 ± 1.1	0.8
M1	Distant lymph nodes	8.3 ± 8.3	6.9 ± 3.5	1.0	5.1 ± 4.3	2.6 ± 1.1	1.0	12.7		8.5 ± 6.3	1.0	4.3	1.7 ± 1.5	1.0
	Bone	15.2 ± 4.7	3.3	0.5	**9.3 ± 5.9**	1.6	0.5	10.9 ± 5.9		2.8	0.5	**2.7 ± 2.3**	0.9	1.0
	Peritoneum	7.6 ± 6.3			**7.5 ± 6.6**			6.5 ± 2.5				**1.7 ± 0.7**		
	Liver	3.1			1.6			5.3				1.7		
	Adrenal	8.6			4.9			11.4				3.5		
	Soft tissue	**7.6**			**3.2**			**42.3**				**14**		
	Lung		5.5			2.2				6.9			1.2	

Data are presented as mean ± SD (median and min-max are provided in supplementary material: supplementary Table 3). Bold highlight percentage differences between [ ^68^ Ga]Ga-FAPI-46 and [ ^18^ F]FDG uptake ≥ 50% for each involved region according to the expression of hormone receptors (supplementary material: supplementary Table 4).

## Discussion

In this small exploratory cohort, the head-to-head comparison showed similar uptake patterns in [^68^Ga]Ga-FAPI-46- and [^18^F]FDG-positive breast cancer lesions of 13 patients with hormone receptor-positive and/or HER2-positive breast cancer. Overall, semi-quantitative comparison of lesion uptake on [^68^Ga]Ga-FAPI-46 and [^18^F]FDG PET/CT across the entire cohort, revealed no significant differences in the mean number of detected lesions, the mean total tumour volume, and the total tumour SUV_mean_ (Table 2).

Differences in detection rates of [^18^F]FDG and FAP-targeted tracers across luminal A/B and HER2-positive breast cancer subtypes could reflect their distinct molecular targets and the specific features of the tumour microenvironment. [^18^F]FDG primarily captures glucose metabolism through GLUT-mediated uptake and hexokinase activity, thereby imaging glycolytic activity (Warburg effect); while FAP tracers bind to CAFs traducing stromal remodelling and desmoplasia [33–35]. FAP is mainly secreted by adipose stromal CAFs, which are more prevalent in luminal A tumours, whereas HER2-positive, luminal B, and triple-negative cancers typically exhibit a more fibrotic stromal architecture [17, 36]. Some authors speculate that, in breast cancer, tumour-surrounding adipocytes may act as key precursors of CAFs, with breast cancer cells altering adjacent adipocytes, leading to reduced lipid content and up-regulation of fibroblast markers (including FAP) [36].

As FAP tracers mark a different biological process, they may complement [^18^F]FDG, especially in low-FDG-avid tumours (such as luminal A, and to a lesser extent luminal B) [33, 37]. However, available data remain heterogeneous: some studies report high FAP uptake in luminal A and HER2-positive subtypes [18], whereas other describe relatively low uptake in luminal A and B subtypes [38]. Although mapping different mechanisms, within our cohort, comparable uptake of [^68^Ga]Ga-FAPI-46 and [^18^F]FDG was observed across luminal-like and HER2-positive breast cancer cases. This is consistent with few previous reports showing no substantial differences in FAP uptake regardless of breast cancer histopathological characteristics, including immunohistochemistry, molecular features, and tumour grade [14, 18, 35, 39]. While we observed non-inferiority of [^68^Ga]Ga-FAPI-46 compared to [^18^F]FDG, a limited number of studies described superiority of using FAP-targeted tracers in breast cancer staging. However, these studies did not identify differences when stratifying by hormone receptor status or HER2 expression. Guo et al. [35] performed a prospective trial with 61 patients, comprising cases of newly diagnosed or suspected breast cancer before initiation of therapy, along with patients presenting with suspected recurrence or metastatic disease. They reported a higher lesion-based detection rate for FAP-targeted tracers compared with [^18^F]FDG, resulting in changes in TNM staging in 22% (13/59) of patients and modifications in clinical management in 15% (9/59). They reported no significant differences in SUV_max_ values derived from either [^18^F]FDG or FAP tracers across receptor-defined subgroups (HR-positive, HR-positive/HER2-positive, HER2-positive, and triple-negative). Median SUV_max_ values for [^68^Ga]Ga-FAPI-46/[^18^F]FAPI-42 compared with [^18^F]FDG in primary breast tumours were consistently higher across all molecular subtypes − 9.6 vs. 2.3 in luminal A, 12.5 vs. 5.0 in luminal B, 12.9 vs. 7.0 in HER2-positive, and 12.4 vs. 3.3 in triple-negative disease. In our study, the median (range) SUV_max_ values for [^68^Ga]Ga-FAPI-46 versus [^18^F]FDG in luminal-like tumours were 9.7 (9.1–10.2) versus 20.8 (4.3–37.3), and in HER2-positive tumours 18.5 (10.3–18.7) versus 9.9 (1.4–11.7). Elboga et al. [18], retrospectively compared [^68^Ga]Ga-FAPI-04 and [^18^F]FDG uptake in 48 patients with breast cancer, irrespective of whether they had received chemotherapy within the previous month. They found that [^68^Ga]Ga-FAPI-04 detected a greater number of lesions and showed higher uptake values than [^18^F]FDG. They also reported no statistical significance related to the breast cancer pathological characteristics. In their study, the following [^68^Ga]Ga-FAPI-04 mean SUV_max_ values were reported in patients with luminal A, luminal B HER2-negative, luminal B HER2-positive, HER-2-enriched, and triple-negative: 10.1 (± 5.6); 11.5 (± 5.4); 21.2 (± 8.3); 17.9 (± 6.0); 11.1 (± 4.1), respectively (*p* = 0.009). They specified that no difference among the luminal subtypes was found [18]. Similarly, in our cohort we found a mean [^68^Ga]Ga-FAPI-46 SUV_max_ of 9.6 (± 0.8) in luminal-like and 15.8 (± 4.8) in HER2-positive patients. In another retrospective study of 19 patients with invasive breast cancer (18 undergoing initial staging and one restaging after treatment for distant metastases), Furthermore, Backhaus et al. [39] found no association between [^68^Ga]Ga-FAPI-46 uptake and tumour grade, receptor status, or histological subtype.

Moreover, the known difficulty in diagnosing cerebral and peritoneal carcinomatosis by [^18^F]FDG, may be in favour of using FAP, mainly due to its low physiological background uptake in the brain and intestines [40–43].

Having in mind the small sample size, in which the uptake patterns may be heavily influenced by individual patients, limiting generalizability, our findings support the use of [^68^Ga]Ga-FAPI-46 across all subgroups due to comparable uptake patterns in [^68^Ga]Ga-FAPI-46- and [^18^F]FDG-positive breast cancer lesions, alongside several practical advantages. These include a more patient-friendly protocol - eliminating the need for pre-imaging preparation (such as fasting for at least 4 h, and resting in a warm and quiet environment for 60 min before [^18^F]FDG PET/CT [32]) and allowing for a shorter interval between tracer injection and image acquisition -as well as promising theranostic applications in combination with radioligand therapy [13, 25]. FAPI-46 contains a DOTA-chelator which enables binding of Ga-68, Y-90, or Lu-177 and it is one of the most common agents used for therapy purposes [44]. The theranostic field is rapidly evolving, with significant future developments anticipated, including the use of peptide-based compounds (^68^Ga/^177^Lu-FAP-2286) and dimeric FAPI derivatives (^68^Ga/^177^Lu-DOTAGA.(SA.FAPi)₂), which are expected to offer longer tumour retention times and, consequently, higher absorbed doses in therapeutic applications [45, 46]. Several FAP tracers with different chemical structure, binding affinity, and internalization rates, have been investigated with the aim to achieve better performance in imaging and therapy purposes [47]. Currently, [^68^Ga]GaFAPI-04 and − 46 are the most used FAP tracers worldwide [47]. Considering that [^68^Ga]-labelling has limitations related to its shorter half-life and restricted synthesis capacity, [^18^F]- or [^99m^Tc]-labelled FAPI compounds ([^18^F]-FAPI-74 for PET, [^99m^Tc]Tc-FAPI-34 for SPECT imaging) have also been explored [48–50]. These compounds enable wider clinical use because of the isotopes longer half-life [44]. Other promising methods to improve FAP biodistribution include albumin binding, targeting peptides or peptidomimetic compounds (such as OncoFAP, BiOncoFAP, FAP-2286 [23, 45, 51–53]) and modifying the quinoline-based structure (ex. DOTA.(SA.FAPi)2 and DOTAGA.(SA.FAPi)2 [13, 46, 54]).

In the coming years, diagnostic work-up is likely to be further complemented by subtype-specific tracers such as [^18^F]FES or radiolabelled anti-HER2 agents, each of which may provide distinct additional diagnostic value in breast cancer [9–11, 55, 56]. Nevertheless, their availability and cost still limit its use in clinical practice. These tracers can only be used in specific histological subtypes, contrary to FAP tracers which may evaluate different tumour regions within the same patient, making it more universal for staging, even at initial diagnosis (and prior to histology).

We acknowledge that the limitations of this study are related to its small sample size that severely limits statistical power and generalizability, lack of histopathological confirmation, follow-up information and impact evaluation on clinical management, as well as a potential selection bias arising from the referral pathway.

## Conclusion

In this small exploratory cohort, the head-to-head comparison of [^68^Ga]Ga-FAPI-46 and [^18^F]FDG PET/CT in hormone receptor and/or HER2-positive breast cancer suggests comparable uptake patterns in [^68^Ga]Ga-FAPI-46- and [^18^F]FDG-positive breast cancer lesions among all subtypes. These results may motivate future research of FAP theranostics in patients with breast cancer.

## Supplementary Information

Supplementary Material 1

## Data Availability

Complementary information and analysis are provided as supplementary material. The datasets used and/or analysed during the current study are available from the corresponding author on reasonable request.

## Abbreviations

[^68^Ga]FAPI: Fibroblast activation protein inhibitor labelled with gallium-68
[^18^F]FDG − 2: [^18^F]fluoro-2-deoxy-D-glucose labelled with fluoride-18
HER2: Human epidermal growth factor receptor 2
SUV: Standardized uptake value
TLR: Tumour-liver ratio
UICC: Union for International cancer control
