# Supplementary material for: Comparison between [68Ga]Ga-FAPI-46 and [18F]FDG PET/CT uptake in luminal-like vs. HER2-positive breast cancer
Source: EJNMMI Res. 2026 Mar 13;16:58. doi: 10.1186/s13550-026-01390-3 (PMC13066216; doi:10.1186/s13550-026-01390-3)
Supplement: Supplementary file 1 — Supplementary Material 1 [file 13550_2026_1390_MOESM1_ESM.docx]

**Supplementary material**

Table 1. Receptor status, HER2 and Ki-67 per patient

| **Patient nº** | **ER +** | **PR +** | **HER2** | **Ki67** | **Luminal A, B or HER2-positive** |
| --- | --- | --- | --- | --- | --- |
|  | 1 | 1 | 0 | 30-35% | B |
|  | 1 | 0 | 1+ | 20-80% | HER2 |
|  | 1 | 1 | 0 | 5% | A |
|  | 1 | 0 | 2+ | 25% | B |
|  | 1 | 1 | 0 | 25% | B |
|  | 1 | 0 | 1+ | 61% | HER2 |
|  | 1 | 1 | 1+ | 50% | HER2 |
|  | 1 | 0 | 0 | 80% | B |
|  | 1 | 1 | 3+ | 20% | HER2 |
|  | 1 | 1 | 1+ | N/A | HER2 |
|  | 1 | 1 | 0 | 5% | A |
|  | 1 | 1 | 1+ | 10-15% | HER2 |
|  | 1 | 1 | 1+/0 | 15%/5% | HER2/A |

Table 2. [^68^Ga]Ga-FAPI-46 and [^18^F]FDG uptake per involved region

| Involved regions | | **[^68^Ga]Ga-FAPI-46** | | | **[^18^F]FDG** | | | **Exclusive FAPI/FDG**  **uptake** |
| --- | --- | --- | --- | --- | --- | --- | --- | --- |
|  |  | Nº pts | SUV_max_ | TLRpeak | Nº pts | SUV_max_ | TLRpeak |  |
| Breast | | 5 | 10.3  (9.1-18.7) | 4.2  (3.5-11.3) | 5 | 9.9  (1.4-37.3) | 2.2  (1.0-14.0) |  |
| Local lymph nodes | | 5 | 7.4  (2.7-20.3) | 2.6  (0.7-5.1) | 6 | 5.3  (1.1-18.9) | 0.7  (0.0-3.3) | 2 FAPI only  3 FDG only |
| M1 | Distant lymph nodes | 5 | 6.7  (2.4-14.2) | 2.1  (1.1-8.2) | 3 | 12.7  (4.0-12.9) | 2.8  (0.7-4.3) | 2 FAPI only |
|  | Bone | 5 | 16.6  (3.3-18.2) | 3.2  (1.6-15.0) | 5 | 7.2  (2.8-14.6) | 1.4  (0.9-4.3) |  |
|  | Peritoneum | 4 | 8.6  (1.5-14.0) | 6.9  (2.3-14.9) | 3 | 6.5  (4.0-8.9) | 1.8  (1.0-2.3) | 1 FAPI only |
|  | Lung | 1 | 5.5 | 2.2 | 1 | 6.9 | 1.2 |  |
|  | Liver | 1 | 3.1 | 1.6 | 1 | 5.3 | 3.8 |  |
|  | Soft tissue | 1 | 7.6 | 3.2 | 1 | 42.3 | 14.0 |  |
|  | Adrenal gland | 1 | 8.6 | 4.9 | 1 | 11.4 | 3.5 |  |
|  |  |  |  |  |  |  |  | p-value |
| Nº of lesions | | 5.0  (1.0-54.0) | | | 5.0  (1.0-156.0) | | | 0.462 |
| Total tumour volume | | 30.2 mL  (0.4-236.9) | | | 27.7 mL  (0.4-421.3) | | | 0.440 |
| Total tumour SUVmean | | 5.3  (0.4-12.1) | | | 3.8  (0.1-23.6) | | | 0.355 |

*Data are presented as median and minimum-maximum. Grey cells highlight percentage difference between [^68^Ga]Ga-FAPI-46 and [^18^F]FDG uptake ≥ 50% for each involved region (supplementary material: table 4).*

Table 3. Comparison of [^68^Ga]Ga-FAPI-46 and [^18^F]FDG uptake per involved region according to the expression of hormone receptors (luminal-like vs HER2-positive)

| Involved regions | | **[^68^Ga]Ga-FAPI-46 uptake** | | | | | | **[^18^F]FDG uptake** | | | | | |
| --- | --- | --- | --- | --- | --- | --- | --- | --- | --- | --- | --- | --- | --- |
|  |  | Luminal  SUV_max_ | HER2  SUV_max_ | p-value | Luminal  TLR_peak_ | HER2  TLR_peak_ | p-value | Luminal  SUV_max_ | HER2  SUV_max_ | p-value | Luminal  TLR_pea_k | HER2  TLRpeak | p-value |
| Breast | | 9.7  (9.1-10.2) | 18.5  (10.3-18.7) | 0.2 | 4.1  (4.0-4.2) | 5.0  (3.5-11.3) | 0.8 | 20.8  (4.3-37.3) | 9.9  (1.4-11.7) | 1.0 | 7.5  (1.0-14-0) | 2.2  (2.0-3.3) | 1.0 |
| Local lymph nodes | | 10.5 | 7.4  (2.7-20.3) | 1.0 | 5.1 | 2.6  (1.1-4.7) | 1.0 | 7.8  (1.1-18.9) | 4.8  (3.4-6.1) | 1.0 | 0.6  (0.0-3.3) | 1.5  (0.7-2.2) | 0.8 |
| M1 | Distant lymph  nodes | 8.3  (2.4-14.2) | 6.9  (4.4-9.4) | 1.0 | 5.2  (2.1-8.2) | 2.6  (1.8-3.4) | 1.0 | 12.7 | 8.5  (4.0-12.9) | 1.0 | 4.3 | 1.8  (0.7-2.8) | 1.0 |
|  | Bone | 17.5  (9.8-18.2) | 3.3 | 0.5 | 9.6  (3.2-15.0) | 1.6 | 0.5 | 13.9  (4.1-14.6) | 2.8 | 0.5 | 2.7  (1.1-4.3) | 0.9 | 1.0 |
|  | Peritoneum | 7.2  (1.5-14.0) |  |  | 7.2  (1.5-14.0) |  |  | 6.5  (4.0-8.9) |  |  | 1.8  (1.0-2.3) |  |  |
|  | Liver | 3.1 |  |  | 1.6 |  |  | 5.3 |  |  | 1.7 |  |  |
|  | Adrenal | 8.6 |  |  | 4.9 |  |  | 11.4 |  |  | 3.5 |  |  |
|  | Soft tissue | 7.6 |  |  | 3.2 |  |  | 42.3 |  |  | 14 |  |  |
|  | Lung |  | 5.5 |  |  | 2.2 |  |  | 6.9 |  |  | 1.2 |  |

*Data are presented as median and minimum-maximum. Grey cells highlight percentage difference between [^68^Ga]Ga-FAPI-46 and [^18^F]FDG uptake ≥ 50% for each involved region (supplementary material: table 4).*

Table 4. Percentage difference between [^68^Ga]Ga-FAPI-46 and [^18^F]FDG uptake

| Region | **All patients** | | | **Luminal-like** | | | **HER2-positive** | | |
| --- | --- | --- | --- | --- | --- | --- | --- | --- | --- |
|  | FAP Mean | FDG Mean | %diff FAP/  FDG | FAP Mean | FDG Mean | %diff FAP/  FDG | FAP Mean | FDG Mean | %diff FAP/  FDG |
| Breast SUVmax | 13.4 | 12.9 | 0.04 | 9.6 | 20.8 | -0.54 | 15.8 | 7.7 | 1.05 |
| Breast TLRpeak | 5.6 | 4.5 | 0.24 | 4.1 | 7.5 | -0.45 | 6.6 | 2.5 | 1.64 |
| Local LN SUVmax | 9.1 | 7.5 | 0.21 | 10.5 | 8.9 | 0.18 | 10.1 | 4.8 | 1.10 |
| Local LN TLRpeak | 2.9 | 1.2 | 1.33 | 5.1 | 1.1 | 3.64 | 2.8 | 1.5 | 0.87 |
| Distant LN SUVmax | 7.4 | 9.9 | -0.25 | 8.3 | 12.7 | -0.35 | 6.9 | 8.5 | -0.19 |
| Distant LN TLRpeak | 3.3 | 2.6 | 0.27 | 5.1 | 4.3 | 0.19 | 2.6 | 1.7 | 0.53 |
| Bone  SUVmax | 13.1 | 8.5 | 0.54 | 15.2 | 10.9 | 0.39 | 3.3 | 2.8 | 0.18 |
| Bone  TLRpeak | 6.3 | 2 | 2.15 | 9.3 | 2.7 | 2.44 | 1.6 | 0.9 | 0.78 |
| Peritoneal SUVmax | 8.2 | 6.5 | 0.26 | 7.6 | 6.5 | 0.17 | NA | NA | NA |
| Peritoneal TLRpeak | 7.7 | 1.7 | 3.53 | 7.5 | 1.7 | 3.41 | NA | NA | NA |
| Liver  SUVmax | 3.1 | 5.3 | -0.42 | 3.1 | 5.3 | -0.42 | NA | NA | NA |
| Liver  TLRpeak | 1.6 | 1.7 | -0.06 | 1.6 | 1.7 | -0.06 | NA | NA | NA |
| Total PET volume | 71.5 | 73.2 | -0.02 | 88.6 | 117.2 | -0.24 | 17.9 | 15 | 0.19 |
| Total SUVmean | 5.4 | 5.4 | 0.00 | 5.5 | 7 | -0.21 | 6.3 | 4.4 | 0.43 |
| Total nº lesions | 14.7 | 18.9 | -0.22 | 18 | 28.7 | -0.37 | 4.6 | 6.8 | -0.32 |

*Grey cells highlight percentage difference between [^68^Ga]Ga-FAPI-46 and [^18^F]FDG uptake ≥ 50% for each involved region. NA – not applicable.*
